# Single balloon enteroscopy-guided endoscopic retrograde pancreatography for the treatment of a symptomatic pancreatic pseudocyst complicated by pancreaticojejunostomy stricture: A case report

**DOI:** 10.1097/MD.0000000000031293

**Published:** 2022-10-28

**Authors:** Eunae Cho, Chang-Hwan Park, Seo Yeon Cho

**Affiliations:** a Division of Gastroenterology, Department of Internal Medicine, Chonnam National University School of Medicine, Gwangju, South Korea.

**Keywords:** altered gastrointestinal anatomy, case report, endoscopic retrograde, enteroscopy, pancreatic pseudocyst, pancreatography

## Abstract

Endoscopic treatment of a pancreatic pseudocyst complicated by pancreaticojejunostomy (PJ) stricture is challenging.

**Patient concerns:**

A 76-year-old woman presented with worsening abdominal pain and dyspepsia. She had been receiving adjuvant chemotherapy (capecitabine and cisplatin) for 4 months after pylorus-preserving pancreaticoduodenectomy (PPPD) for the treatment of extrahepatic cholangiocarcinoma.

**Diagnoses:**

Laboratory findings included elevated serum amylase (145 U/L) and lipase (437 U/L) levels. Abdominal computed tomography (CT) showed a pancreatic pseudocyst of approximately 3 cm in size and pancreatic duct dilatation in the remnant pancreas. According to the Response Evaluation Criteria in Solid Tumors, cholangiocarcinoma is a stable disease.

**Interventions and outcomes:**

Endoscopic drainage of the pancreatic pseudocyst was planned. Single-balloon enteroscopy (SBE)-guided endoscopic retrograde pancreatography (ERP) with endoscopic ultrasonography (EUS) using a mini probe demonstrated a membranous PJ stricture and a pancreatic pseudocyst. Endoscopic pseudocyst drainage using a 7-Fr plastic stent was successfully performed after needle-knife incision of the PJ stricture. Follow-up abdominal CT after 3 weeks showed complete resolution of the pseudocyst. Chemotherapy was resumed.

**Lessons:**

SBE-guided ERP with EUS using a mini probe may be an effective and safe treatment in a patient with a pancreatic pseudocyst complicated by membranous PJ stricture after PPPD.

## 1. Introduction

Pancreaticoduodenectomy (PD) is the primary surgical treatment for cancers involving the pancreatic head, distal common bile duct, and duodenum.^[1]^ Delayed gastric emptying and pancreatic fistulas are the most common complications of PD and pancreaticojejunostomy (PJ) stricture.^[2]^ PJ stricture can cause abdominal pain, recurrent acute pancreatitis, and pseudocyst.^[2,3]^ The main treatment of PJ stricture includes endoscopic drainage or surgical revision.^[4,5]^ Surgical revision of PJ stricture is complex and is associated with high morbidity up to 26%; thus, surgeons may hesitate to perform additional surgery for this benign disease.^[5]^ In turn, endoscopic drainage is preferred in most cases. Traditionally, endoscopic drainage of PJ stricture is performed via endoscopic retrograde pancreatography (ERP).^[4]^ Because ERP after PD is challenging, low technical and clinical success rates have been reported.^[6]^ Herein, we report a case in which a pancreatic pseudocyst complicated by membranous PJ stricture was successfully treated via short-type single-balloon enteroscopy (SBE)-guided ERP and endoscopic ultrasonography (EUS) using a mini probe.

## 2. Case presentation

A 76-year-old woman from the oncology department was referred to the gastroenterology department with epigastric pain and dyspepsia that had started 1 month ago and had worsened over the last 2 weeks. Physical examination showed tenderness in the epigastric area. She denied having fever or chills. Nine months ago, she was diagnosed with extrahepatic cholangiocarcinoma with lymph node metastasis. After 3 months of neoadjuvant chemotherapy with gemcitabine, cisplatin, and albumin-bound paclitaxel, she underwent laparoscopic pylorus-preserving PD with R1 resection. After pylorus-preserving PD, she had been receiving capecitabine and cisplatin as adjuvant chemotherapy in the oncology department for 4 months. Three weeks ago, chemotherapy was stopped because of aggravated abdominal pain and poor oral intake.

**Figure 1.**
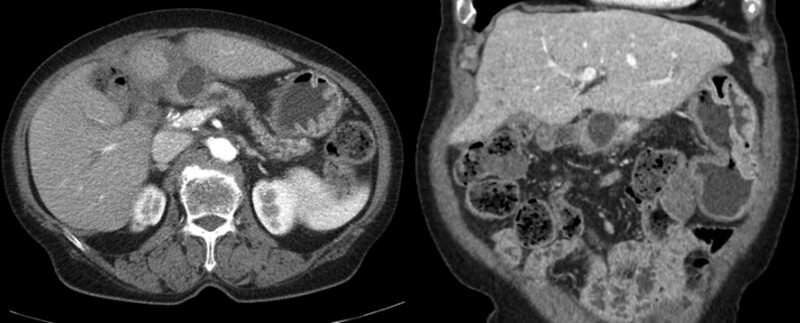
Abdominal computed tomography showed a pancreatic pseudocyst of 3 cm in size and dilated pancreatic duct of the remnant pancreas.

Laboratory findings were as follows: white blood cell count, 8000/µL (neutrophils 80.9%); hemoglobin, 8.8 g/dL; platelet, 160,000/µL; aspartic acid aminotransferase, 27 (10–37) IU/L; alanine aminotransferase, 46 (10–37) IU/L; alkaline phosphate, 548 (35–129) IU/L; total bilirubin, 0.77 (0.2–1.3) mg/dL; amylase, 145 (0–100) U/L; lipase, 437 (7–60) U/L; C-reactive protein, 13.47 (< 0.3) mg/dL; and carbohydrate antigen 19–9 31.2 (0–37) U/mL. Abdominal computed tomography (CT) revealed a mild dilatation of intrahepatic bile ducts, a newly appearing 3-cm-sized pancreatic pseudocyst, and pancreatic duct dilatation in the remnant pancreas (Fig. 1). No definitive evidence of cholangiocarcinoma recurrence was observed, and it was considered a stable disease according to the Response Evaluation Criteria in Solid Tumors.

**Figure 2.**
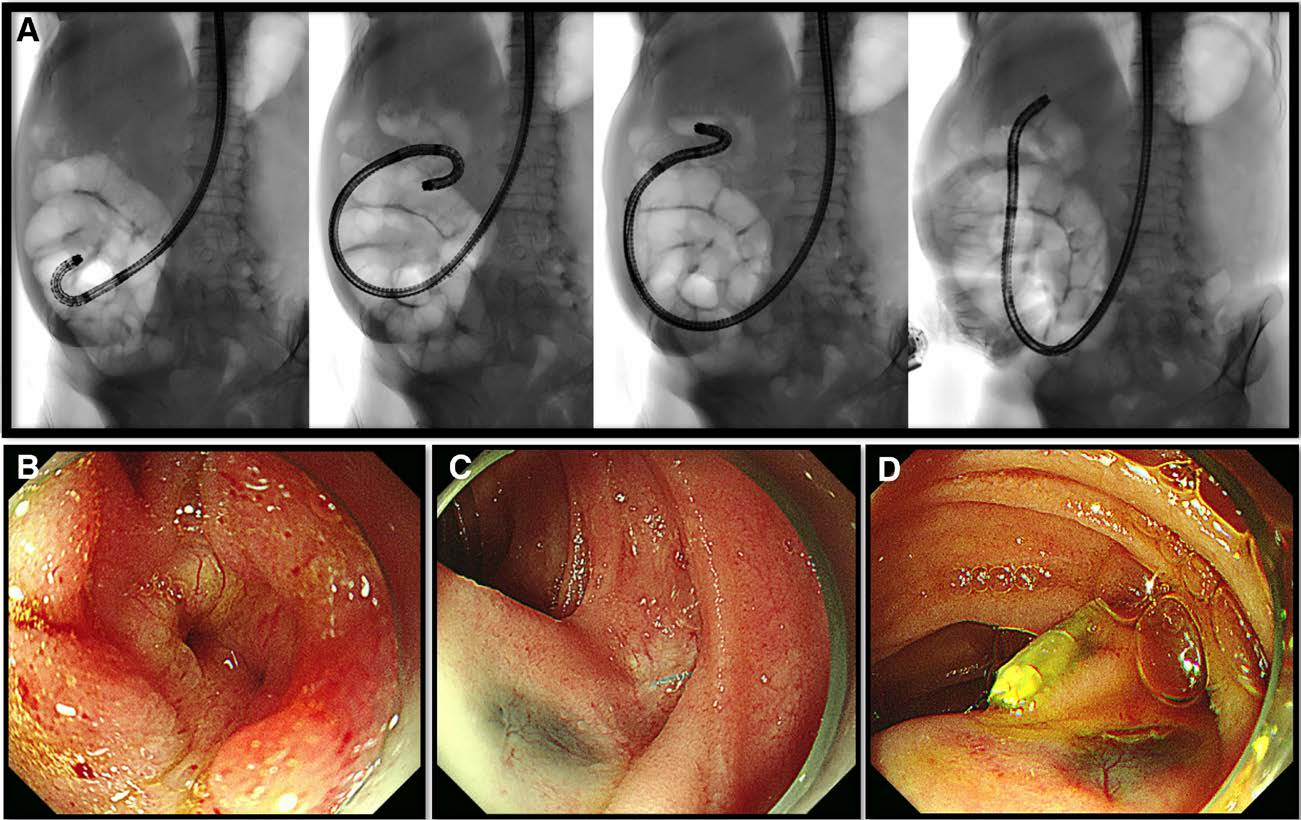
Short-type single-balloon enteroscopy. (A) Fluoroscopic view of the single-balloon enteroscope intubation into the blind loop. (B) Irregular mucosa at the hepaticojejunostomy site. (C) A thin membrane at the suspected pancreaticojejunostomy site. (D) An endoclip was attached near the suspected pancreaticojejunostomy site.

**Figure 3.**
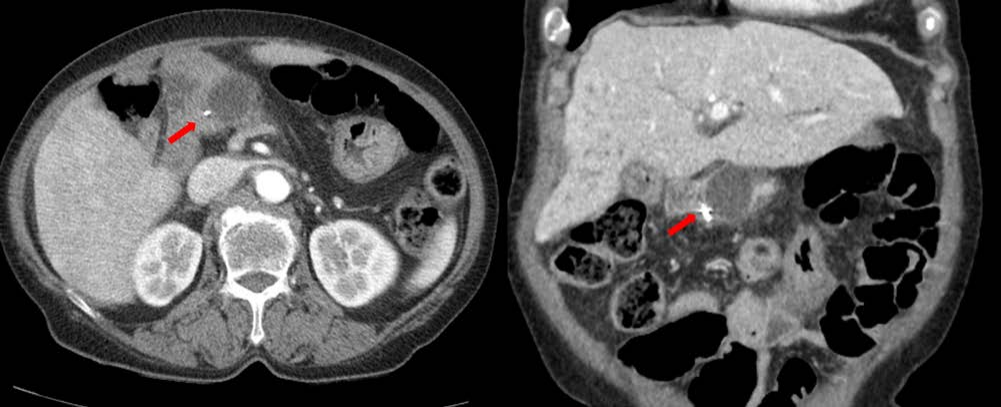
Abdominal computed tomography showed the increased size (3.5 cm) of the pancreatic pseudocyst and an endoclip (red arrow) next to it.

**Figure 4.**
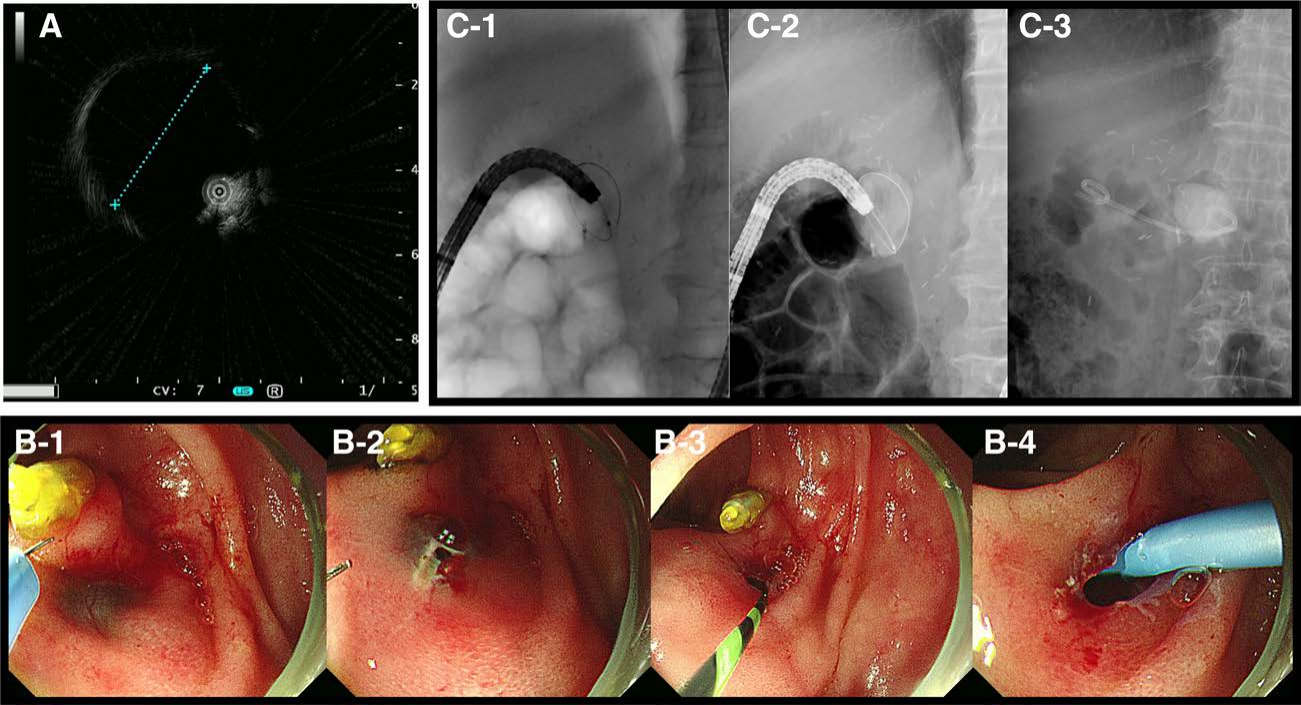
Single-balloon enteroscopy-guided endoscopic retrograde pancreatography. (A) Endoscopic ultrasonography using a mini probe showed a 3.5-cm cystic lesion below the pancreaticojejunostomy (PJ) anastomotic site. (B) Endoscopic and (C) flurosocopic views of the procedure. (B-1) After puncturing the membrane at the PJ anastomotic site, (B-2) pancreatic juice gushed out. (B-3 and C-1) Cannulation into the pseudocyst was performed using a guidewire and a catheter. (C-2) Dye was injected, and (B-4 and C-3) a 7-Fr 7-cm double pigtail plastic stent was deployed. PJ = pancreaticojejunostomy.

**Figure 5.**
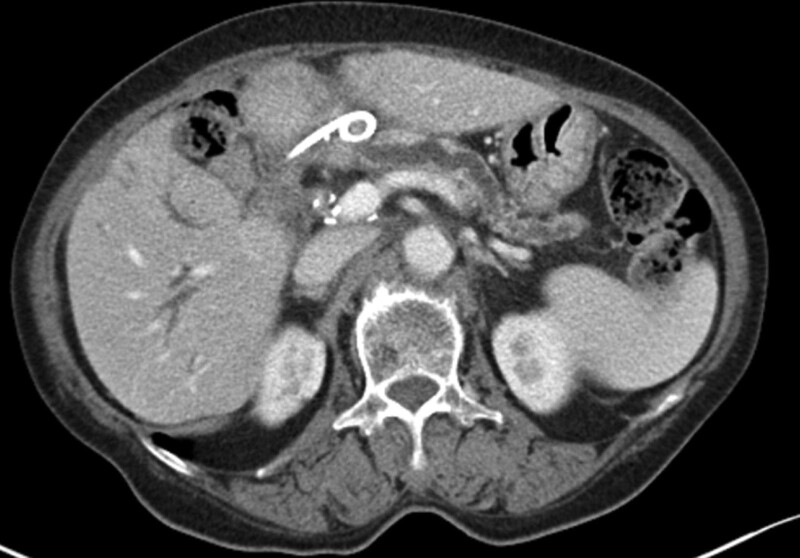
Follow-up abdominal computed tomography 3 weeks after the endoscopic treatment showed a plastic stent and resolution of the pancreatic pseudocyst.

We assumed that the worsening abdominal pain resulted from the pancreatic pseudocyst complicated by the PJ stricture. Therefore, endoscopic drainage of the pancreatic pseudocyst was planned. A short-type SBE (SIF-H290S, Olympus Medical Systems, Tokyo, Japan) was used for intubation to the blind end of the jejunum (Fig. 2A). Forceps biopsy was taken from irregular mucosa at the hepaticojejunostomy (HJ) anastomotic site (Fig. 2B). A thin transparent membrane-like structure was noted at the suspected PJ anastomotic site (Fig. 2C). Cannulation of the pancreatic duct was impossible because of complete obstruction by the membranous structure. To ensure the exact location of PJ anastomosis, an endoclip was placed next to the suspected PJ anastomotic site (Fig. 2D). Abdominal CT showed the endoclip right next to the pseudocyst, whose size increased to 3.5 cm (Fig. 3). Two days later, a second session SBE-guided ERP was performed. After finding the PJ anastomotic site and the endoclip, EUS was performed using a mini probe to demonstrate the 3.5-cm anechoic lesion at the PJ anastomotic site (Fig. 4A). After puncturing the membrane-like structure with a needle knife (Microknife XL, Boston Scientific, MA), clear fluid gushed out from the opening (Figure B and Supplementary video, Supplemental Digital Content, http://links.lww.com/MD/H762). After cannulation of the pseudocyst with a 0.035-inch guidewire (Jagwire, Boston Scientific) and a catheter (MTW Endoskopie Manufaktur, Wesel, Germany), the obtained pancreatogram confirmed the pseudocyst and dilated pancreatic duct (Fig. 4B and C). Endoscopic drainage using a 7-Fr 7-cm double pigtail plastic stent (Boston Scientific) was successfully performed (Fig. 4B and C). No adverse events associated with the procedure occurred. On the next day, the patient was discharged without symptoms. Histology of the biopsy at the HJ anastomotic site revealed well-differentiated adenocarcinoma. Follow-up abdominal CT after 3 weeks showed complete resolution of the pseudocyst (Fig. 5), and the patient remained asymptomatic. Chemotherapy was resumed, and the patient received 6 more months of chemotherapy without tumor progression.

## 3. Discussion

PJ stricture is a challenging late complication after PD.^[7]^ Because the most common indications of PD were pancreatic head cancer or distal cholangiocarcinoma in the past, the overall survival time of the patients was not long enough to unveil PJ stricture as a complication after PD. However, nowadays, with the advancements in chemotherapy and surgical techniques and broader indications of PD such as chronic pancreatitis and pancreas cystic neoplasm, the average time to develop PJ stricture after PD is 12 to 46 months and the occurrence rate ranges from 2% to 11.3%.^[7,8]^ PJ stricture has been recognized to cause persistent abdominal pain, recurrent acute pancreatitis, and pseudocysts.^[3,4]^

Endoscopic treatment of PJ stricture has been appealing to endoscopists. However, ERP after PD is challenging. First, it is difficult to access the afferent limb and identify PJ anastomosis with standard endoscopes because of the long length of the afferent limb, postoperative strictures, and acute angulation. Recently, SBE- or double-balloon enteroscopy (DBE)-guided ERP has increased successful intubation rates up to 71% to 97.5%.^[9–11]^ Although DBE may provide much better outcomes, SBE enables less preparation time and better maneuverability than DBE.^[12]^ Additionally, short-type SBE with a shorter length and wider working channel allows the utilization of almost all conventional instruments for ERP.^[13]^ Therefore, we used short-type SBE in the present case, and intubation into the afferent limb and identification of HJ and PJ anastomosis sites were successful within a relatively short time (approximately 20 min).

Then, cannulation and treatment of the PJ stricture are other challenges in patients with PD. Even with the use of balloon enteroscopy including SBE or DBE, technical success ranges from 23.1% to 76%.^[11,14,15]^ Therefore, endoscopic ultrasound-guided pancreatic duct drainage (EUS-PDD) techniques have been developed. The three techniques of EUS-PDD include the rendezvous technique, transenteric antegrade stenting, and transmural stenting.^[16]^ Compared with the traditional ERP approach, EUS-PDD has the advantage of higher technical success rates ranging from 72% to 92.5%.^[4,15]^ However, it is associated with higher complication rates than the traditional ERP approach. In a multicenter study comparing EUS-PDD with enteroscopy-guided ERP after Whipple surgery, adverse events were more common in the EUS-PDD group (35% vs 2.9%, *P* = .001).^[15]^ A systematic review summarized more adverse events with EUS-PDD than with traditional ERP.^[4]^ A wide range of adverse events from EUS-PDD included abdominal pain, bleeding, pancreatitis, perforation, pancreatic abscess, guidewire shearing, fever, pneumoperitoneum, pseudocyst, and perigastric fluid collection.^[17]^ As such, the current guidelines recommend EUS-PDD as a salvage procedure after failed ERP or an alternative to enteroscopy-guided ERP in patients with surgically altered anatomy.^[18]^

Finally, it is a real challenge to treat a symptomatic pseudocyst complicated by PJ stricture. Current guidelines recommend EUS-guided transmural drainage or transpapillary drainage for pseudocysts adjacent to the stomach or duodenum.^[19,20]^ However, no consensus is established yet regarding the treatment of pseudocysts complicated by PJ strictures. A symptomatic pseudocyst complicated by PJ stricture might be treated with various nonsurgical methods including percutaneous catheter, EUS-, and enteroscopy-guided drainage. Abdominal CT of the current case showed that a pseudocyst was located at the PJ site, which was right below the left hepatic lobe and at least 4 cm off from the stomach (Fig. 1). Although percutaneous drainage of the pseudocyst might be considered, a risk of permanent pancreaticocutaneous fistula could not be underestimated. Additionally, the puncture site of EUS-guided pseudocyst drainage was too far from the stomach. Generally, the optimal puncture site is the point where the distance is the shortest between the cyst and the gut wall (usually < 1 cm) and without intervening vessels or other organs.^[21]^ Therefore, we performed enteroscopy-guided drainage. EUS with a mini probe was also planned to determine the exact location of the pseudocyst complicated by the PJ stricture.

Enteroscopy-guided ERP for the treatment of complicated PJ stricture has several advantages. It is more physiological than EUS-guided interventions. No new pancreatoenteric fistulas have to be made. It also has lower complication rates than EUS-guided interventions.^[4,15]^ Furthermore, both HJ and PJ anastomosis sites can be examined. By directly examining HJ and PJ anastomosis sites, biopsies can be performed, and the cause of HJ or PJ stricture – whether it is a benign or a recurred malignancy – can be evaluated. In the current case, a biopsy was taken at the HJ site and revealed well-differentiated adenocarcinoma. Precise diagnosis by histology may lead to a significant change in further treatment and may affect the patient’s survival.

In summary, a symptomatic pancreatic pseudocyst complicated by PJ stricture was treated by SBE-guided ERP with EUS using a mini probe. EUS using a mini probe played the main role in delineating the needle-knife puncture site by viewing the underlying pseudocyst and PJ site anastomosis. Moreover, SBE helped in detecting cholangiocarcinoma at the HJ anastomosis site. The resolution of the pseudocyst by endoscopic drainage and early detection of cholangiocarcinoma made it possible to continue adjuvant chemotherapy. Short-type enteroscopy-guided ERP with EUS using a mini probe could be considered an effective and safe treatment modality in patients with a pancreatic pseudocyst complicated by PJ stricture.

## Author contributions

**Conceptualization**: Chang-Hwan Park

**Methodology**: Eunae Cho

**Project administration**: Eunae Cho, Seo Yeon Cho

**Resources**: Eunae Cho

**Writing–original draft**: Eunae Cho

**Writing–review & editing**: Chang-Hwan Park

## Supplementary Material


